# Exploring the Lived Experiences of Adults With Sickle Cell Disease in Nigeria Using van Manen's Phenomenological Approach

**DOI:** 10.1002/puh2.70148

**Published:** 2025-11-05

**Authors:** Florence Idowu Michael, Chinomso Nwozichi, Omolabake Salako, Mosidat Oshodi‐Bakare, Elizabeth Olaogun, Josiah Nang‐Bayi

**Affiliations:** ^1^ Department of Nursing, School of Nursing Babcock University Ilisan Remo Ogun State Nigeria; ^2^ Wellstar School of Nursing Kennesaw State University Kennesaw Georgia USA; ^3^ Oncology Nursing Society of Nigeria Ilisan Remo Ogun State Nigeria; ^4^ Sunyani SDA Hospital Sunyani Ghana

**Keywords:** lived experiences, Nigeria, phenomenology, sickle cell disease

## Abstract

**Purpose:**

This study explores the lived experiences of adults with sickle cell disease (SCD) in Ilorin, Kwara State, Nigeria, providing insights into their struggles and coping mechanisms.

**Design:**

A qualitative phenomenological study was conducted to gain a deeper understanding of the lived experiences of adults with SCD.

**Methods:**

This study employed a phenomenological approach to explore the lived experiences of adults with SCD. Semi‐structured interviews were conducted with 24 participants, selected through purposive sampling. Data were analyzed thematically using van Manen's phenomenological approach to capture the depth of participants’ experiences.

**Findings:**

Findings revealed that adults with SCD endure recurrent pain crises, emotional distress, stigmatization, and substantial financial burdens. Participants reported difficulties in accessing specialized healthcare, leading to increased reliance on alternative treatment methods. Social support was often inadequate, with many experiencing isolation and discrimination. Coping strategies identified included spiritual reliance, engagement in peer support networks, and self‐management practices.

**Conclusions:**

The study underscores the urgent need for comprehensive healthcare interventions, improved psychosocial support systems, and public health policies tailored to the unique challenges faced by adults with SCD. Addressing these issues through multidisciplinary approaches can enhance the quality of life for individuals living with SCD in Nigeria and similar settings.

**Clinical Evidence:**

The findings highlight critical areas for healthcare improvement, including the need for accessible specialized care, financial support mechanisms, and community‐driven psychosocial interventions. Integrating culturally relevant coping strategies, such as spiritual support and peer networks, into healthcare services may improve patient outcomes and overall well‐being.

## Graphical abstract

This study explores the lived experiences of Nigerian adults with sickle cell disease using van Manen's phenomenological approach. Findings reveal profound physical, temporal, spatial, and relational burdens, highlighting the urgent need for integrated care, psychosocial support, stigma reduction, and culturally grounded interventions in low‐resource settings.

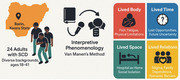

## 1 Introduction

Sickle cell disease (SCD) is a hereditary blood disorder that affects millions worldwide. It is caused by a mutation in the beta‐globin gene, leading to the production of abnormal hemoglobin (HbS), which causes red blood cells to become rigid and sickle‐shaped [1]. These deformed cells obstruct blood flow, resulting in severe pain episodes, organ damage, and increased mortality. SCD predominantly affects individuals of African, Mediterranean, Middle Eastern, and Indian descent, with sub‐Saharan Africa accounting for the highest global burden [1, 2]. Nigeria has one of the largest population of individuals with SCD, with approximately 150,000 children born with the disease each year [3], making it a major public health concern. SCD impacts 1%–3% of populations across African countries, accounting for a significant 7%–16% of deaths in children under five [4].

SCD significantly impacts the socioeconomic well‐being of Nigerian families by affecting guardians’ employment and income, increasing healthcare costs and time spent in hospitals, and ultimately diminishing their quality of life [5]. SCD imposes a heavy physical burden through chronic pain and complications, and a significant psychological burden from depression, collectively worsening patients’ quality of life and contributing to increased healthcare use and substance abuse [6]. Despite medical advancements, many affected individuals in Nigeria and other low‐resource settings lack access to adequate healthcare [7], leading to poor disease management and reduced life expectancy [8]. The transition from pediatric to adult healthcare presents additional challenges for individuals living with SCD, as specialized care becomes less accessible, and adult patients often struggle with maintaining treatment regimens due to financial and social constraints [1, 9].

Adults with SCD face a unique set of physical, psychological, and socioeconomic challenges. The disease significantly impacts their quality of life, limiting their ability to engage in education, employment, and social activities [5, 10]. Many individuals experience stigmatization in their communities due to misconceptions about the disease. In some cases, cultural beliefs contribute to social exclusion, with individuals being viewed as weak or cursed [11]. Furthermore, the chronic nature of the illness places a financial burden on patients and their families, exacerbating health disparities in resource‐limited settings.

Although research on SCD in children has been extensive [12], there is a significant gap in the literature regarding the lived experiences of adults with the disease, particularly in African contexts. Applying van Manen's phenomenology allows this study to move beyond clinical perspectives and capture the subjective realities of individuals navigating the challenges of adulthood with SCD. By understanding these experiences through the lens of existential phenomenology, this study seeks to provide insights that can inform policy development and improve healthcare support for this vulnerable population.

## 2 Methods

### 2.1 Theoretical Lens

This study utilized a phenomenological approach, which is particularly suited to exploring individuals’ lived experiences and uncovering the essence of their perceptions. Within this framework, van Manen's interpretive phenomenology [13, 14] was applied to explore the meaning‐making process of adults with SCD. This approach allowed the researcher to understand SCD as it was lived, experienced, and articulated by participants themselves. The goal was to identify the core domains and patterns that characterized the lived experience of adults with SCD.

### 2.2 Participants and Setting

Participants were recruited from hematology clinics and SCD support groups in Ilorin, Kwara State, Nigeria. Recruitment strategies included collaboration with healthcare professionals at Kwara State University Teaching Hospital, and utilizing word‐of‐mouth referrals from existing participants as recommended by Creswell and Creswell [15]. Inclusion criteria included being an adult (aged 18 and above) diagnosed with SCD and willing to share their lived experiences. A purposive sampling strategy was employed to select 24 participants, ensuring diverse perspectives in terms of age, socioeconomic background, and disease severity. Although recruiting primarily from clinics and support groups might introduce a selection bias, it ensured that participants had received some form of medical care and could articulate their experiences.

### 2.3 Data Collection

Data were collected through semi‐structured, in‐depth interviews conducted in private settings to ensure confidentiality and encourage openness. Sociodemographic information (age, gender, marital status, educational level, employment status, primary caregiver, and religious affiliation) were collected at the beginning of each interview session. Each interview lasted approximately 40–60 min and was audio‐recorded with participants’ consent. The interview guide included open‐ended questions designed to explore participants’ experiences with physical health, emotional well‐being, social interactions, financial impact, and coping mechanisms. For instance, key questions included: “What has it been like for you, in your own words, to live with sickle cell disease here in Nigeria?” and “Can you describe a time when a sickle cell crisis was particularly challenging for you – what was that experience like?” Observational field notes were also taken to ensure the validity of the responses during analysis. In line with phenomenological traditions, interviews were conversational and fluid, allowing participants to narrate their stories in their own words and at their own pace.

Data analysis proceeded using thematic analysis guided by van Manen's interpretive phenomenological approach [13, 14]. An inductive approach was employed, where initial codes were generated directly from the data. Codes were then refined, categorized, and grouped into broader domains. The research team engaged in rigorous discussions and debates to ensure inter‐rater reliability and refine the coding framework. This was primarily achieved through regular meetings where two independent researchers, working collaboratively, reviewed and categorized the transcribed interview data, with any discrepancies resolved through consensus discussions involving the wider research team. Key domains emerged, representing core aspects of participants’ lived experiences of SCD. These domains were further developed and refined through a process of constant comparison and cross‐checking with raw data, allowing the essence of their experiences to emerge organically.

### 2.4 Rigor

Rigor was demonstrated through an iterative process of data collection and analysis. For instance, after conducting the first five interviews, a recurring theme emerged regarding the dismissal of pain by healthcare providers. This insight led to a refinement of subsequent interview questions to further explore participants’ perceptions of empathy and trust in clinical encounters. Methodological triangulation was achieved by integrating multiple data sources, including interview transcripts and observational field notes gathered during clinic visits and community meetings. These field notes captured behaviors such as long wait times and visible discomfort in clinic settings, which helped contextualize participant narratives.

Reflexivity was maintained through continuous self‐reflection. The lead researcher kept a reflexive journal throughout the study, recording evolving assumptions and emotional reactions. One entry, for example, revealed an assumption that participants would prioritize biomedical explanations for their pain. This was challenged when several participants attributed their symptoms to spiritual causes, prompting the research team to broaden the conceptual framework used during analysis. These reflections were discussed regularly in team meetings to check for potential bias in interpretation.

Member checking was conducted with a subset of six participants selected to represent diverse backgrounds in terms of age, gender, and care‐seeking experiences. Participants reviewed a summary of the preliminary findings. One participant clarified that her hesitancy in seeking care was not rooted in cultural beliefs, as initially interpreted, but rather in prior negative encounters with healthcare professionals. This feedback led to a revision of the corresponding theme. Peer debriefing sessions were held with two qualitative research experts outside the immediate team. Their feedback prompted the refinement of themes, particularly the consolidation of overlapping ideas related to endurance and faith into a broader category of adaptive meaning‐making. An audit trail was maintained to document coding decisions, theme development, and analytical shifts. These measures ensured transparency and strengthened the trustworthiness of the findings by grounding them in the participants’ lived experiences while accounting for potential researcher bias.

## 3 Result

### 3.1 Sociodemographic Characteristics

A total of 24 participants (Table 1), aged 18–41 years, were included in this study, with an equal representation of males (50%) and females (50%). Most participants were single, whereas a few were married. Their educational backgrounds varied, including high school, undergraduate, and graduate‐level education. Employment status was diverse, with participants identifying as students, employed, self‐employed, or unemployed. The majority relied on family members [1] for primary caregiving, whereas others were supported by their spouses [4] or cared for themselves [16]. Religious affiliation was split between Islam and Christianity.

**TABLE 1:** Sociodemographic data of participants.

Participant ID	Age (years)	Gender	Marital status	Educational level	Employment status	Primary caregiver	Religious affiliation
1	26	Female	Single	High school	Student	Family member	Islam
2	34	Female	Single	Undergraduate	Student	Self	Christianity
3	18	Female	Single	High school	Unemployed	Family member	Islam
4	36	Female	Married	Graduate	Self‐employed	Self	Christianity
5	29	Male	Single	Undergraduate	Employed	Family member	Islam
6	27	Male	Single	High school	Unemployed	Family member	Islam
7	31	Male	Single	Undergraduate	Unemployed	Family member	Christianity
8	19	Male	Single	High school	Student	Family member	Islam
9	39	Male	Married	Graduate	Unemployed	Spouse	Christianity
10	23	Male	Single	High school	Unemployed	Family member	Islam
11	20	Female	Single	High school	Self‐employed	Family member	Islam
12	30	Female	Single	Undergraduate	Student	Family member	Islam
13	33	Male	Single	Undergraduate	Employed	Self	Christianity
14	21	Male	Single	High school	Employed	Family member	Islam
15	22	Female	Single	High school	Student	Family member	Islam
16	40	Female	Married	Graduate	Self‐employed	Spouse	Christianity
17	35	Male	Single	Undergraduate	Unemployed	Self	Christianity
18	38	Female	Married	Graduate	Student	Spouse	Christianity
19	41	Male	Married	Graduate	Employed	Spouse	Christianity
20	25	Male	Single	High school	Employed	Family member	Islam
21	28	Female	Single	Undergraduate	Self‐employed	Family member	Islam
22	32	Female	Single	Undergraduate	Self‐employed	Self	Christianity
23	37	Male	Married	Graduate	Employed	Self	Christianity
24	24	Female	Single	High school	Self‐employed	Family member	Islam

The teams and subthemes are categorized on the basis of the van Manen phenomenological framework (Table 2).

**TABLE 2:** Themes and subthemes.

Dimension	Theme	Subtheme
Lived body (corporeality)	Pain and physical limitations	Chronic pain and fatigue
		Unpredictability of symptoms
		Physical vulnerability and limitations
	Coping with pain and symptoms	Medication and hydration
		Heat therapy and isolation
		Alternative medicine
Lived time (temporality)	Disrupted life trajectory	Educational and career interruptions
		Delayed personal milestones
	Fear of the future	Uncertainty of health outcome
		Concerns over financial stability
	Time as a constraint and burden	Time lost to illness
		Awareness of mortality
Lived space (spatiality)	Hospital as a second home	Dependence on medical spaces
		Positive and negative experiences with healthcare
	Restrictions on movement and activities	Physical constraints
		Fear of environmental triggers
	Home as a site of care and isolation	Protective household environment
		Self‐imposed isolation
Lived relations (relationality)	Family as primary support system	Parental sacrifices
		Overprotection and dependency
	Social stigma and misunderstanding	Peer isolation
		Discrimination in schools and workplaces
	Romantic and marriage challenges	Reduced prospects
		Family pressure
	Community perception and support	Lack of awareness
		Support from online community

## 4 Lived Body (Corporeality)—The Physical Experience of the Body

The lived body refers to how individuals experience their own bodies, particularly in relation to health and illness. For individuals living with SCD, the body is often perceived as unpredictable, vulnerable, and a site of pain.

### 4.1 Theme 1: Pain and Physical Limitations

Participants described how the pain is not just a physical burden but also affects their mental well‐being, their independence, and their interactions with others. The unpredictability of pain crisis forces individuals to be constantly on guard, making it difficult to plan ahead or lead a normal life. Many participants struggle with physical vulnerability, feeling restricted in their ability to engage in normal physical activities. This results in frustration, dependency, and social isolation.

#### 4.1.1 Subtheme 1.1: Chronic Pain and Fatigue

Pain crises are an unavoidable and painful aspect of living with SCD. Participants spoke about how the pain often starts suddenly, affecting various parts of their body such as their legs, chest, and back. The fatigue that comes with these crises is persistent and draining, leaving individuals feeling exhausted even when they are not experiencing acute pain.

One participant described how the pain could strike unexpectedly, even in crucial moments:

Sometimes, I will be writing [an] exam and then suddenly crash, fall sick. The pain is so much I just want to disappear. It is not something you [can] explain to people who don't have it. (Participant 6)

#### 4.1.2 Subtheme 1.2: Unpredictability of Pain Crisis

One of the most distressing aspects of SCD is the unpredictability of pain crises. Participants expressed fear and frustration at the inability to anticipate when a crisis might occur, making it difficult to plan for their future or even manage their daily activities. The lack of control over their bodies creates anxiety and stress.

One participant voiced the difficulty of dealing with an uncertain future:

I don't know what [will] happen next. Yesterday I was fine, today I am in bed. Tomorrow, maybe the hospital. There is no way to predict it. (Participant 3)

For some, even a careful routine does not guarantee stability:

I used to think that if I took my drugs and rested, I would be fine. But this sickness does not listen. It does what it wants, when it wants. (Participant 9)

#### 4.1.3 Subtheme 1.3: Physical Vulnerability and Limitations

Physical limitations such as difficulty walking long distances, limited ability to lift heavy objects, and doing basic house chores running or doing basic house chores, due to SCD prevent individuals from fully participating in normal activities, leading to frustration and feelings of inferiority. Many participants described how their bodies do not allow them to do what their peers can, making them feel left out or different from others.

One participant described the restrictions imposed by their condition:

I can't do what my mates are doing. They run, they play, they dance. Me? I sit [and] watch. It hurts to be left out, but what can I do? My body cannot carry [me]. (Participant 17)

Another participant described how basic tasks can feel overwhelming:

Even lifting a bucket of water feels like carrying a mountain. My body refuses to cooperate [with me]. I want to be independent, but I always need help. (Participant 8)

### 4.2 Theme 2: Management of Pain and SCD Symptoms

Living with SCD necessitates various coping mechanisms to manage chronic pain and unpredictable crises. Participants described different strategies they employ to alleviate pain and maintain some control over their condition. These coping methods range from medical treatments to traditional remedies and personal techniques developed over time. Managing SCD is a delicate balance of adhering to medical advice, avoiding triggers, and finding what works best for individual needs.

#### 4.2.1 Subtheme 2.1: Medication and Hydration

Many participants rely on routine medications, painkillers, and hydration to manage their symptoms. Some reported strict adherence to prescribed drugs, whereas others admitted reluctance due to side effects. Medications help in reducing the intensity of pain, but hydration is equally crucial in preventing crises.

One participant described how their family plays an essential role in ensuring continuous access to medication:

My parents make sure my drugs are always available. Before I finish one, another is waiting. They say I must always have medicine because any delay can bring [on] a crisis. It's like my life depends on those tablets and injections. (Participant 2)

Another participant emphasized the importance of water intake:

I take plenty of water. Water is life for me. I don't joke with my drugs [either]. If I forget to drink enough water, I start feeling weak, and before I know it, I am in pain. So, I carry my water bottle everywhere. (Participant 19)

#### 4.2.2 Subtheme 2.2: Heat Therapy and Isolation

Several participants mentioned using heat therapy to relieve pain, often isolating themselves during crises. Applying heat helps soothe aching muscles, whereas isolation provides a way to endure pain without external disturbances. Many find comfort in withdrawing from social interactions when pain becomes overwhelming.

A participant described how heat therapy provides some relief during episodes of pain:

I wrap myself in a blanket, use hot water, and stay in one place. No movement, no noise. That is the only way I can manage the pain when it comes suddenly. If I move too much, the pain gets worse. (Participant 9)

Isolation is not just a coping mechanism for some, it is also a way to avoid judgment from others:

People don't understand why I disappear sometimes. They think I am avoiding them. But the truth is, when I am in pain, I don't have [the] strength to pretend I am fine. I just lock my door and wait for it to stop. (Participant 5)

#### 4.2.3 Subtheme 2.3: Alternative Medicine

A few participants reported using traditional medicine, herbal remedies, or non‐prescribed treatments in their quest to manage SCD symptoms. Although some were uncertain about the effectiveness, they still considered these alternatives as part of their coping strategy.

A participant explained their reliance on herbal treatments despite not being sure of the outcomes:

Sometimes, I drink herbal mixtures. I don't know if they work, but at least I try. My grandmother insists that certain leaves can help with my blood, so I drink it, hoping it will reduce the crises. (Participant 10)

Some participants expressed skepticism about traditional remedies but admitted to trying them in desperate moments:

Honestly, I don't believe in all these herbs, but when the pain is too much, I will drink anything if someone tells me it will help. I just want the pain to stop. (Participant 22)

## 5 Lived Time (Temporality)—The Experience of Time in Relation to Illness

The lived experience of time is altered by illness, pain, and uncertainty. Time is no longer linear or predictable but is instead shaped by crises, recovery, and waiting. Participants frequently described lost opportunities, disrupted life plans, and anxiety about the future. They expressed frustration over the inability to plan ahead, as every aspect of life is dictated by the frequency and severity of their illness.

### 5.1 Theme 1: Disrupted Life Trajectory

SCD creates significant disruptions in the expected timeline of life events. Participants reported setbacks in their education, career progression, and personal milestones, such as marriage and independence. These delays and interruptions often lead to feelings of frustration, inadequacy, and helplessness, as they watch their peers move ahead, whereas they remain stuck in cycles of illness and recovery.

#### 5.1.1 Subtheme 1.1: Educational and Career Interruptions

Many participants struggled through school due to frequent health crises. The unpredictability of their condition makes academic consistency nearly impossible, leading to prolonged years in school or, in some cases, the inability to complete their education.

One participant lamented how their illness has significantly delayed their academic progress:

I should have graduated by now, but the sickness won't let me. Every time I start making progress, another crisis happens, and I have to stop. My mates have finished, some are even working, and I am still here, trying to catch up. (Participant 5)

Another participant described how their education has become secondary to their health struggles:

School is a luxury for me. I attend when I can, but my health decides. Some days, I wake up feeling strong enough to go; other days, I am stuck in bed or in the hospital. Exams, assignments, lectures—I miss so many things. I just take things one step at a time. (Participant 22)

#### 5.1.2 Subtheme 1.2: Delayed Personal Milestones

SCD does not just affect education and career but also interferes with major life events, such as marriage, childbirth, and achieving independence. Participants described how their condition has delayed or prevented them from fulfilling societal and personal expectations.

One participant expressed sadness about watching their younger siblings move ahead, whereas they remain in the same place:

All my younger siblings are married, but me? I am still here, fighting sickness every day. They are building families, moving forward, and I am just trying [to] stay alive. Sometimes I wonder if I will [ever] get that chance. (Participant 11)

#### 5.1.3 Subtheme 1.3: Loss of Independence

The inability to financially support oneself due to frequent hospitalizations and limited job opportunities results in prolonged dependence on family. This dependency can lead to feelings of frustration, helplessness, and guilt.

One participant shared their desire for financial independence:

I want to work, earn [my own] money, and be independent like my mates. But which employer will keep someone [who i]s always calling in sick? So, I rely on my family. It's frustrating because I know I have potential, but my health limits me. (Participant 19)

Another participant described their internal conflict:

I hate asking my parents for money all the time, but I have no choice. Every hospital visit, every drug I need—it all costs money. If I could take care of myself, I would. (Participant 8)

### 5.2 Theme 2: Fear of the Future

For many individuals with SCD, the future is uncertain. The unpredictable nature of the disease, coupled with its potential complications, makes it difficult to have long‐term goals or expectations. Many participants shared concerns about their life expectancy and the fear of what lies ahead.

#### 5.2.1 Subtheme 2.1: Uncertainty of Health Outcome

Participants fear the unpredictability of their condition and the possibility of early death. Living with SCD means constantly navigating between periods of wellness and debilitating illness, making it difficult to plan for the future.

One participant voiced their deep uncertainty about aging with SCD:

Will I even grow old? I don't know. I just take one day at a time and hope for the best. I see people making five‐year plans, ten‐year plans, and I can't even plan for next month because I don't know what will happen. (Participant 4)

#### 5.2.2 Subtheme 2.2: Concerns Over Financial Stability

The high cost of lifelong medical care is a major source of anxiety. Many participants worry about affording treatments, medications, and hospital visits.

One participant described their financial struggles:

I am always thinking about money. Medications, hospital bills, check‐ups—it never ends. Even when I am feeling okay, I know another bill is coming soon. (Participant 9)

Another participant shared their experience with employment difficulties:

I want to work and be independent, but my health keeps getting in the way. How do I save money when I am always in the hospital? (Participant 20)

### 5.3 Theme 3: Time as a Constraint and Burden

Time is perceived as both limited and burdensome by individuals with SCD. Hospital visits, treatments, and health setbacks consume significant portions of their lives, preventing them from fully engaging in personal and professional pursuits.

#### 5.3.1 Subtheme 3.1: Time Lost to Illness

Frequent hospital visits, long treatment sessions, and recovery periods result in substantial time lost that could have been used for education, career, and social activities.

One participant shared their frustration:

I spend so much time in hospitals [that] I feel like I have lost years of my life. While my friends are moving ahead, I am stuck in a cycle of sickness and recovery. (Participant 6)

Another participant reflected on the impact of illness on their goals:

Every hospital admission means missed classes, missed work, missed opportunities. I wonder how much I could have achieved if I didn't have to deal with this. (Participant 15)

#### 5.3.2 Subtheme 3.2: Awareness of Mortality

Many participants reported that living with SCD forces them to reflect on their own mortality, shaping their perspectives on life and purpose.

One participant expressed their awareness of mortality:

Living with this disease makes you think [about] life differently. Every crisis reminds me [that] tomorrow is not promised. I try to make the most of [the] time I have, but it's hard when I feel like I'm running out of it. (Participant 13)

## 6 Lived Space (Spatiality)—The Impact of Illness on One's Environment

Lived space refers to how individuals interact with their physical surroundings. Participants described hospitals, homes, and social environments as both places of care and sites of restriction. For many, the hospital is both a place of relief and an unwelcome second home, while their social lives become restricted due to the fear of triggering painful crises.

### 6.1 Theme 1: Hospital as a Second Home

This theme captures the deep relationship that individuals with SCD develop with medical spaces. Hospitals, initially meant for treatment and care, often become places where they spend significant portions of their lives. Although these spaces provide essential medical interventions, they also foster feelings of dependency, frustration, and emotional exhaustion.

#### 6.1.1 Subtheme 1.1: Dependence on Medical Spaces

Frequent hospital admissions shape the daily lives of individuals with SCD, often making hospitals feel more like home than their actual living spaces.

One participant described their deep familiarity with the hospital setting:

I know the hospital better than [my own] house. Nurses know me by name. I can even tell you the shift of some of the doctors because I have been here [hospital] so many times. (Participant 13)

Another participant reflected on how hospital stays disrupt their sense of normalcy:

I feel like I live in two places…home and the hospital. But the truth is, I spend more time here [hospital] than anywhere else. I see the walls of the hospital more than I see my own bedroom. (Participant 9)

#### 6.1.2 Subtheme 1.2: Positive and Negative Experiences With Healthcare

While some participants found comfort in the hospital setting, others experienced frustration due to unsympathetic healthcare providers or the emotional toll of repeated admissions.

One participant saw the hospital as a place of refuge:

As much as I hate being here [hospital], this is the only place where I know I will get help. At home, I try to endure the pain, but when it gets too much, the hospital is the only option. (Participant 6)

However, another participant expressed exhaustion:

Every time I am admitted, I lose a little bit of myself. I just want to live a normal life, but the hospital keeps pulling me back in. (Participant 20)

### 6.2 Theme 2: Restrictions on Movement and Activities

Living with SCD requires constant vigilance over physical activities, as overexertion or exposure to environmental triggers can lead to painful crises. Participants described feeling confined by these restrictions, as they are unable to participate freely in everyday activities.

#### 6.2.1 Subtheme 2.1: Physical Constraints

SCD places physical limitations on movement and participation in social life. Many participants described avoiding certain environments or activities out of fear of triggering a crisis.

One participant described their self‐imposed limitations:

I avoid going out. I don't want stress that will land me in [the] hospital. I have seen what happens when I push [myself] too much, so I just stay in my comfort zone. (Participant 7)

#### 6.2.2 Subtheme 2.2: Fear of Environmental Triggers

Participants expressed constant concern about exposure to environmental factors that could provoke a crisis, leading them to avoid cold weather, physical exertion, and stressful situations.

One participant spoke about their need to avoid extreme temperatures:

The cold is my enemy. The moment the weather changes, I start feeling pain. So, I stay indoors as much as I can. (Participant 15)

Another participant emphasized the importance of stress management:

If I get too stressed, I know a crisis is coming. I have to be extra careful with work, school, even family arguments. My health is too fragile to take risks. (Participant 19)

### 6.3 Theme 3: Home as a Site of Care and Isolation

Home, which should be a place of comfort and freedom, is often transformed into a site of medical care and imposed restrictions for individuals with SCD. Family members play an active role in enforcing rules meant to protect their health, but these measures can sometimes lead to feelings of confinement.

#### 6.3.1 Subtheme 3.1: Protective Household Environment

Many families take active measures to protect individuals with SCD by limiting their activities and ensuring they avoid situations that might lead to a crisis.

One participant appreciated their family's vigilance:

My mother always reminds me to drink water, rest, and avoid stress. She watches over me like a hawk. I know she means well, but sometimes it feels like I have no freedom. (Participant 10)

#### 6.3.2 Subtheme 3.2: Self‐Imposed Isolation

In addition to family‐imposed restrictions, some participants described withdrawing from social interactions out of fear of judgment, pity, or vulnerability.

One participant shared their experience of avoiding social situations:

I stopped attending parties and gatherings. People always ask questions or look at me with pity. It's easier to just stay home and avoid it all. (Participant 12)

Another participant explained how fear of health decline contributes to their isolation:

I have missed out on so many things because I was afraid of getting sick. My friends have fun, go on trips, and I stay back because I know I can't take the risk. (Participant 17)

## 7 Lived Relations (Relationality)—Social and Interpersonal Impact of Illness

This dimension explores how illness affects social interactions, relationships, and societal attitudes. Participants shared how SCD influences their interactions with family, friends, and romantic partners. Although family is often the primary source of support, the condition also brings challenges such as social stigma, workplace discrimination, and relationship difficulties.

### 7.1 Theme 1: Family as Primary Support System

For many individuals with SCD, family serves as their main source of support, providing emotional, financial, and physical care. Although families offer comfort, their protective nature can also lead to dependency and restrictions on independence.

#### 7.1.1 Subtheme 1.1: Parental Sacrifices

The financial and emotional burden of caring for someone with SCD weighs heavily on families, especially parents, who must make sacrifices to ensure the well‐being of their loved ones.

One participant highlighted their parents’ sacrifices:

My parents sacrifice a lot for me. I live because of them. From buying my medications to rushing me to the hospital in the middle of the night, they do everything. I sometimes wonder how they cope with all the stress. (Participant 16)

Another participant described how the financial strain affects their family:

My parents work extra jobs just to pay for my medical bills. I feel guilty sometimes because I know it's not easy for them. (Participant 14)

#### 7.1.2 Subtheme 1.2: Overprotection and Dependency

Many participants described feeling restricted by well‐meaning but controlling family care. Although these protective measures are meant to safeguard their health, they often lead to frustration and a lack of autonomy.

One participant shared their experience:

My mother treats me like I am made of glass. She doesn't let me do anything on my own. I appreciate her care, but sometimes, I just want to feel like a normal person. (Participant 7)

Another participant expressed frustration over their dependency:

I wish I could do things on my own, but my family is always watching me, making sure I don't ‘overdo’ anything. It makes me feel weak, even when I am not. (Participant 12)

### 7.2 Theme 2: Social Stigma and Misunderstanding

SCD is often misunderstood, leading to stigma and discrimination in social settings. Participants reported experiencing exclusion from peers, schools, and workplaces due to misconceptions about their illness.

#### 7.2.1 Subtheme 2.1: Peer Isolation

Some participants lost friendships due to stigma surrounding SCD. Misconceptions about the disease, including assumptions that those with SCD are weak or will not live long, lead to social rejection.

One participant recounted how childhood friends distanced themselves:

They stopped talking to me. They said I was weak, that I was going to die soon. It hurt because these were people I grew up with, but they saw me as different and didn't want to be around me anymore. (Participant 18)

Another participant shared their struggle:

People think I am pretending or looking for sympathy when I say I am in pain. Over time, I just stopped explaining myself. (Participant 9)

#### 7.2.2 Subtheme 2.2: Discrimination in Schools and Workplaces

Many participants described being excluded from academic or professional opportunities due to their condition.

One participant shared their experience at school:

Some teachers don't understand why I miss school so often. I once overheard a teacher say I was using my sickness as an excuse to be lazy. (Participant 11)

Another participant recounted difficulty securing employment:

I have lost job opportunities because they assume I will always be sick. Even when I explain that I can manage my condition, they don't want to take the risk of employing me. (Participant 20)

### 7.3 Theme 3: Romantic and Marriage Challenges

Participants reported difficulties in relationships due to the fear of passing on SCD to their children or concerns about their life expectancy. Many faced rejection from romantic partners or their families.

#### 7.3.1 Subtheme 3.1: Reduced Prospects

Fear of rejection due to genotype status prevents many individuals with SCD from forming long‐term relationships.

One participant explained their experience:

When I tell someone I have sickle cell, they immediately assume the worst. Some people don't even want [to] continue the conversation. (Participant 15)

Another participant described their fear of relationships:

I have been in love before, but when we started talking about the future, he asked, ‘What if we have [a] child with SCD?’ I didn't have an answer. I can't promise I won't pass this on, and that scares people away. (Participant 8)

#### 7.3.2 Subtheme 3.2: Family Pressure

Some families discourage relationships due to concerns about passing on the sickle cell gene, making it difficult for individuals with SCD to find partners.

One participant shared their experience:

He loved me, but his mother said I would die young, so he left. She told him not to marry me because I wouldn't survive long enough to build a life with him. That broke me. (Participant 20)

Another participant expressed frustration with family expectations:

My family keeps reminding me that I should [only] marry someone with the AA genotype. But love is not always [that] straightforward. (Participant 17)

### 7.4 Theme 4: Community Perception and Support

Community attitudes toward SCD vary widely, with many misconceptions affecting how individuals with the condition are treated. Some participants experienced misunderstanding and stigma, while others found solace in online communities.

#### 7.4.1 Subtheme 4.1: Lack of Awareness

Many communities lack proper understanding of SCD, leading to harmful stereotypes and superstitions.

One participant described their frustration:

People say all kinds of things about sicklers. Some think it is a curse or that it [is] contagious. There is so much ignorance out there. (Participant 21)

Another participant spoke about religious misconceptions:

Some people tell me to pray more, that my faith will heal me. They don't understand that this is [a]medical condition, not something I can just wish away. (Participant 19)

#### 7.4.2 Subtheme 4.2: Support From Online Communities

While physical communities may lack understanding, some participants found support in online groups where they could connect with others experiencing similar challenges.

One participant shared their positive experience:

The only place I feel truly understood is in my online support group. We share experiences, tips, and encouragement. It helps [me] feel less alone. (Participant 10)

Another participant described how online communities provide emotional support:

Whenever I am feeling down, I go to my sickle cell support forum. Talking to others who understand what I am going through makes a big difference. (Participant 6)

## 8 Discussion of Findings

This phenomenological study explored the lived experiences of adults with SCD in Ilorin, Kwara State, Nigeria, revealing significant challenges in physical, psychological, social, and financial domains. The findings align with and expand upon existing literature, demonstrating both universal and context‐specific aspects of living with SCD.

### 8.1 Lived Body (Corporeality)—The Physical Experience of the Body

This study reveals that adults with SCD in Nigeria primarily experience their bodies as sources of unpredictable chronic pain and profound limitation, directly impacting their ability to engage in daily activities, maintain employment, and pursue education. This consistent experience of incapacitating pain, fatigue, and the resulting dependency and social isolation aligns with existing literature [17, 18]. Furthermore, the unpredictability of symptoms creates significant anxiety, making long‐term planning difficult and fostering feelings of restriction. Unlike findings from high‐income countries that emphasize pharmacological pain management and concerns about opioid dependency [17], our participants often reported limited access to adequate pain relief, leading to a greater reliance on varied coping strategies, including non‐medical interventions and, crucially, isolation to manage suffering [19].

The research significantly adds to the body of knowledge by highlighting the exacerbated psychological and social burdens of SCD within a Nigerian context. Beyond the well‐documented anxiety and depression, participants frequently described profound feelings of isolation stemming from stigma, a finding consistent with other African and Caribbean studies [10]. Uniquely, the cultural framing of SCD as a “curse” or “punishment” [11, 20] intensifies internalized stigma and social exclusion, setting it apart from contexts where such beliefs are less prevalent. Moreover, although the concept of “time toxicity” [21] and lost life opportunities resonated, a notable distinction was the participants’ predominant reliance on spiritual coping mechanisms and faith‐based resilience rather than formal institutional support or disability accommodations, a finding consistent with other Nigerian studies on chronic disease management [20]. This underscores the critical role of culturally specific coping strategies and the distinct challenges faced in low‐resource settings.

### 8.2 Lived Time (Temporality)—The Experience of Time in Relation to SCD

This study highlights how adults with SCD in Nigeria experience a profound disruption in their perception of time, with their lives largely shaped by illness‐related interruptions. Participants consistently reported setbacks in education, career progression, and personal milestones like marriage and financial independence due to frequent hospitalizations and physical limitations. This led to pervasive feelings of frustration, inadequacy, and a sense of falling behind peers, aligning with findings that emphasize SCD's disruptive impact on educational and occupational trajectories [22, 23]. The uncertainty of health outcomes further compounded fears about the future and a persistent awareness of mortality, pushing many to live day by day, consistent with previous research on the psychological burden of SCD [24].

This research significantly contributes to the body of knowledge by uniquely contrasting the temporal experiences of individuals with SCD in Nigeria with those managing other chronic illnesses. Unlike some studies on Type 1 diabetes [25] and cancer [26, 27] where patients develop proactive time management or redefine goals to maximize short‐term quality of life, participants with SCD in this study predominantly perceived time as lost and constrained by their illness. This finding suggests a less adaptive or proactive temporal orientation compared to individuals with other chronic conditions like Multiple Sclerosis who engage in adaptive goal‐setting [28]. The insights gained here underscore the distinct challenges faced by individuals with SCD in Nigeria, emphasizing the need for interventions that not only address physical and psychological burdens but also explore and foster strategies for reclaiming agency and reframing their experience of temporality in the face of persistent unpredictability.

### 8.3 Lived Space (Spatiality)—The Impact of SCD on One's Environment

Participants described hospitals as both places of refuge and sites of emotional exhaustion. Although hospitals provided necessary medical interventions, they also symbolized dependency and disruption of normal life. Many participants expressed frustration over the frequency of hospital admissions, and the emotional toll it took on their sense of normalcy. This finding is supported by research from Childerhose et al. [17] which found that individuals with SCD often experience conflicting emotions about hospital care, relying on it for relief while resenting its recurrent necessity.

Beyond medical settings, participants faced restrictions in their social environments. The need to constantly monitor physical activity, avoid environmental triggers, and limit strenuous activities led to self‐imposed isolation. Many feared exposing themselves to conditions that might trigger painful crises, which constrained their ability to participate in everyday social interactions. Home was also perceived as a space of both care and restriction, with family members enforcing protective measures that, although well‐intentioned, often led to feelings of confinement. Studies by Buscetta et al. [19] and Koelbel et al. [22] corroborate these findings, illustrating that individuals with SCD frequently modify their lifestyles to minimize health risks, often leading to social withdrawal.

### 8.4 Lived Relations (Relationality)—Social and Interpersonal Impact of SCD

This study highlights the complex and often challenging social and interpersonal impact of SCD on adults in Nigeria. Although family members provide crucial support, their protective tendencies can inadvertently foster over‐dependence and limit autonomy, mirroring findings from other studies [24, 29]. A pervasive theme was the profound experience of social stigma and misunderstanding, leading to isolation from peers, loss of friendships, and workplace discrimination. Participants recounted rejections in romantic relationships due to fears of genetic transmission, which is a deeply entrenched issue in many African societies where genetic counseling and awareness are still evolving [16]. This underscores that despite advancements in some contexts, individuals with SCD in Nigeria continue to face significant social rejection, even more acutely than suggested by studies from developed countries where comprehensive counseling might foster greater acceptance [30].

The findings significantly add to the body of knowledge by emphasizing the unique depth of social rejection and the struggle for autonomy experienced by Nigerian adults with SCD. Although the impact on employment and romantic relationships due to health concerns and genetic transmission fears is consistent with existing literature [9, 11, 16], this study accentuates how cultural beliefs about SCD exacerbate these challenges. Crucially, in the face of these formidable barriers, participants found vital solace and a sense of belonging in online support groups. This highlights the increasing importance of virtual communities [31] as a modern coping mechanism and a critical source of emotional and social support, particularly where immediate social circles may be less understanding or accepting. This points to a resilient adaptation in navigating relational challenges within a context where traditional societal structures may fall short.

### 8.5 Limitations

This study has some limitations that should be considered when interpreting the findings. First, the study was conducted in a specific geographic region, which may limit the generalizability of the results to other settings with different healthcare infrastructures and sociocultural contexts. Second, the reliance on self‐reported experiences may introduce recall bias, as participants’ perceptions of past events might be influenced by their current emotional and physical state. Additionally, the sample size, although appropriate for qualitative research, may not capture the full diversity of experiences within the broader SCD population. Future studies could expand the sample size and incorporate longitudinal data to track changes in lived experiences over time.

### 8.6 Recommendations for Further Studies

Future research should explore interventions that enhance quality of life and provide better coping strategies to navigate the challenges associated with SCD. Studies focusing on resilience‐building strategies and adaptive coping mechanisms could offer valuable insights into how individuals with SCD manage their condition over time. Additionally, investigating the role of healthcare accessibility, social support systems, and policy interventions in improving patient outcomes could further inform targeted healthcare interventions. Comparative studies examining the experiences of individuals with SCD across different cultural and healthcare contexts could also provide a broader understanding of the disease's impact globally.

## 9 Conclusion

The lived experiences of individuals with SCD are marked by a complex interplay of physical suffering, social isolation, disrupted life trajectories, and psychological distress. The findings highlight the need for more holistic healthcare approaches that address both the medical and psychosocial aspects of SCD. Improved public awareness, employer accommodations, and support systems can help mitigate the social and emotional burdens faced by individuals living with the condition. Future research should explore interventions that enhance quality of life and provide better coping strategies to navigate the challenges associated with SCD. These findings align with previous studies, whereas offering deeper insight into how SCD affects various aspects of life, emphasizing the necessity of multi‐faceted support systems for affected individuals.

## Author Contributions

**Florence Idowu Michael**: conceptualization, methodology design, data collection, data analysis, manuscript drafting, and overall project coordination. **Chinomso Nwozichi**: supervision, conceptualization, methodology refinement, data analysis, critical manuscript review, and final approval. **Omolabake Salako**: data collection, literature review, methodology refinement, data analysis, critical manuscript review, and final approval. **Mosidat Oshodi‐Bakare**: data collection, literature review, data analysis, manuscript drafting, editing, and contribution to interpretation of results. **Elizabeth Olaogun**: data collection, literature review, assistance with data interpretation, manuscript drafting, and critical revision for intellectual content. **Josiah Nang‐Bayi**: literature review, assistance with data interpretation, critical manuscript review.

## Funding

The authors received no specific funding for this work.

## Ethics Statement

Ethical approval for this study was obtained from the Babcock University Health Research Ethics Committee (BUHREC). All procedures performed in studies involving human participants were in accordance with the ethical standards of the institutional research committee and with the 1964 Helsinki declaration and its later amendments.

## Consent

Informed consent was obtained from all individual participants included in the study. Participants were informed about the purpose, risks, and benefits of the study, and their rights to withdraw at any time without penalty.

## Conflicts of Interest

The authors declare no conflicts of interest.

## Data Availability

The data supporting the findings of this study are available from the corresponding author upon reasonable request. Due to ethical considerations and confidentiality agreements with participants, raw interview transcripts are not publicly available.
